# Unique *Cryptosporidium* Population in HIV-Infected Persons, Jamaica

**DOI:** 10.3201/eid1405.071277

**Published:** 2008-05

**Authors:** Wangeci Gatei, Donnett Barrett, John F. Lindo, Denise Eldemire-Shearer, Vitaliano Cama, Lihua Xiao

**Affiliations:** * Centers for Disease Control and Prevention, Atlanta, Georgia, USA; † Atlanta Research and Education Foundation, Decatur, Georgia, USA; ‡ The University of the West Indies, Kingston, Jamaica

**Keywords:** Cryptosporidium, cryptosporidiosis, epidemiology, subtype, genotype, HIV, dispatch

## Abstract

A cryptosporidiosis survey showed the presence of *Cryptosporidium hominis*, *C. parvum, C. canis*, and *C. felis* in 25, 7, 1, and 1 HIV-positive persons from Jamaica, respectively; 1 person had both *C. hominis* and *C. felis*. Multilocus sequence typing indicated the presence of a homogeneous but geographically distinct *C. hominis* population in Jamaica.

Cryptosporidiosis is endemic to most tropical countries including Jamaica (*1*). Some evidence suggests that clinical manifestations of cryptosporidiosis may vary according to *Cryptosporidium* species and subtypes (*2**,**3*). Differentiating the species and subtypes with an aim of understanding the transmission dynamics of the parasites in disease-endemic areas requires the use of high resolution molecular tools (*4*). So far, few studies have reported on the molecular epidemiology of cryptosporidiosis in the Caribbean region, and only 2 small-scale studies on *Cryptosporidium* spp. in HIV-infected persons in Haiti have been conducted (*5**,**6*). In this preliminary study, genotyping and multilocus sequence typing (MLST) techniques were used to investigate the transmission of cryptosporidiosis among HIV-infected patients in Jamaica.

## The Study

Stool specimens were obtained from HIV-infected adults in Kingston, Jamaica, as part of routine parasitologic diagnosis. All patient identifiers were removed before specimen acquisition. A total of 35 *Cryptosporidium-*positive stool specimens were collected from May 2003 through July 2007. Only 1 specimen per patient was included in the study. Specimens were collected from multiple hospitals in Kingston; some of the patients were from outlying areas who came to Kingston for medical care. Specimens were stored in 2.5% potassium dichromate at 4°C before analysis. Thereafter, DNA was extracted by using the FastDNA Spin Kit for Soil (Qbiogene Inc., Carlsbad, CA, USA); *Cryptosporidium* spp. were identified on the basis of PCR–restriction fragment length polymorphism analysis of the small subunit ribosomal RNA gene as described (*7*). Identification of *C. hominis* and *C. parvum* subtypes was based on sequence polymorphism at the 60-kDa glycoprotein gene (GP60) locus by using the nomenclature described (*8*). Nineteen of the 25 *C. hominis*–positive specimens and all 7 *C. parvum*–positive specimens were analyzed by MLST. The typing targeted 5 additional loci, including the 47-kDa protein (CP47 microsatellite), a serine repeat antigen (MSC6–7 minisatellite), a hypothetical retinitis pigmentosa GTPase regulator (RPGR minisatellite), and a hydroxyproline-rich glycoprotein (DZHRGP minisatellite and microsatellite) in chromosome 6 and the 70-kDa heat shock protein (HSP70 minisatellite) in chromosome 2. Nomenclature and classification of subtypes at these 5 loci have been described (*9*).

Genotype analysis of the 35 *Cryptosporidium*–positive specimens showed that 25 had *C. hominis*, 7 had *C. parvum*, 1 had *C. canis*, 1 had *C. felis,* and 1 had both *C. hominis* and *C. felis*. Initial subtyping of *C. hominis* and *C. parvum* used sequence analysis of the GP60 gene. Most (22) *C. hominis* specimens had subtype IbA10G2; 3 had subtype IeA12G3T3. All 7 *C. parvum* specimens belonged to subtype IIcA5G3d, which was identical to *C. parvum* identified in children in South Africa (GenBank accession no. AF440636).

Results of the MLST analysis of the additional 5 loci showed limited gene diversity in *C. hominis*. At the CP47 locus, all 19 specimens analyzed belonged to subtype IA39G22. Most of the other loci were also monomorphic, and subtypes at these loci were identified by amplicon sizes. All specimens were 590 bp at the DZHRGP locus, 358 bp at the RPGR locus, and 1,095 bp at the HSP70 locus. At the MSC6–7 locus, 18 of the 19 *C. hominis* specimens analyzed were 494 bp; 1 was 509 bp.

Diversity was more pronounced in the MLST analysis of *C. parvum,* although all specimens belonged to IIcA5G3d subtype at the GP60 locus. There were 3 subtypes at the DZHRGP locus. Subtype 1 was 496 bp and was found in 4 specimens. Subtype 2 was also 496 bp but had 1 single nucleotide polymorphism at position 314; this subtype was seen in 2 specimens. Subtype 3 was 499 bp and was found in 1 specimen. The HSP70 locus had 2 subtypes, differentiated by the presence of a single nucleotide polymorphism (a change of C to T at position 172), with 6 of the 7 specimens in 1 subtype. All *C. parvum* specimens were monomorphic at the CP47, MSC6–7, and RPGR loci. Results of the *C. hominis* and *C. parvum* subtyping at the 5 loci are shown in the Table.

**Table:** Subtypes of *Cryptosporidium hominis* and *C. parvum* at 6 loci identified in specimens from Jamaica*

GP60	CP47	MSC6-7	DZHR-GP	RPGR	HSP70
*Cryptosporidium hominis*, n = 25 (19 used in MLST)					
IbA10G2 (22)	IA39G22 (19)	494 bp (18)	590 bp (19)	358 bp (19)	1095 bp (19)
IeA12G3T3 (3)		509 bp (1)			
*C. parvum*, n = 7 (7 used in MLST)					
IIcA5G3d (7)	IIA24G11C1 (7)	461 (7)	496a bp (4)	391 bp (7)	1059a bp (6)
			496b bp (2)		1059b bp (1)
			499 bp (1)		

*Numbers in parentheses show the frequency of subtypes at each locus. MLST, multilocus sequence typing.

To measure the degree of heterogeneity of *C. hominis* and *C. parvum*, an MLST analysis was conducted to compare MLST types from Jamaica with those from other geographic areas of similar GP60 subtype families Ib, Ie and IIc (Kenya, Peru, India, and USA). All sequences for each specimen at the 5 loci were concatenated and multilocus subtypes generated. The analysis showed *C. hominis* multilocus subtypes from Jamaica clustered in 1 group, regardless of their GP60 subtype designation. Specimens of the same GP60 subtypes from other regions clustered in clades separate from the Jamaica specimens, forming largely distinct monophyletic groups defined by geographic origin. Specimens from Kenya and India clustered in 1 clade because of extensive human migration between the 2 countries. Although all *C. parvum* specimens from Jamaica were identified as IIcA5G3d, 4 MLST types were present, highlighting the extent of genetic diversity of *C. parvum* in this study. The relationship of the multilocus subtypes based on the 5 loci inferred by neighbor-joining analysis is shown in the Figure.

**Figure:**
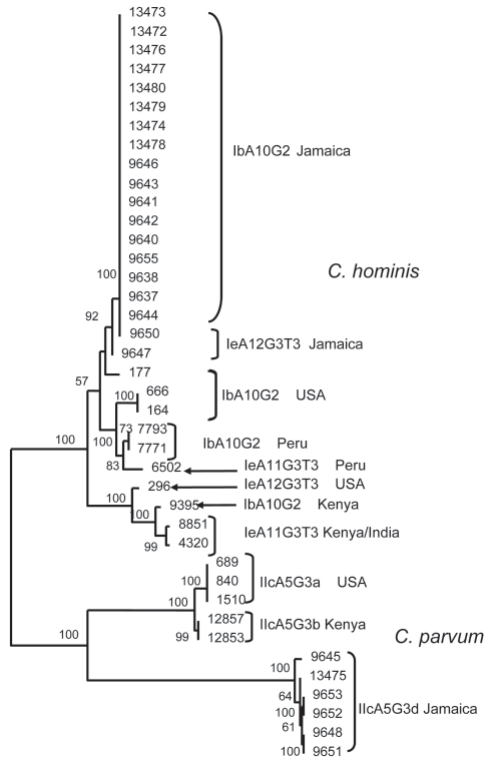
Relationships among *Cryptosporidium hominis* and *C. parvum* multilocus sequence subtypes at 5 genetic loci. Parasite population from Jamaica was compared with that from other regions by neighbor-joining analysis of concatenated sequence of 5 genetic loci, by using GP60 subtype identification in specimen selection. The Kimura 2-parameter model was used in the distance calculations. The sequences reported in this paper are available in the GenBank database under accession nos. EU141710–EU141727.

## Conclusions

Results of our study show that anthroponotic transmission is important in the epidemiology of cryptosporidiosis in Jamaica. This conclusion is based on our finding of *C. hominis* and anthroponotic *C. parvum* (the IIc subtype family) in 33 (94%) of 35 specimens analyzed. Because all *C. parvum–*positive specimens belonged to the anthroponotic subtype family IIc and because 1 of the *C. felis–*infected patients had concurrent infection with *C. hominis*, it is possible that some of the *C. canis* and *C. felis* infections seen in this study were transmitted through anthroponotic pathways.

Results of the GP60 subtyping showed only 2 *C. hominis* subtype families (Ib and Ie); most belonged to the IbA10G2 subtype. Although the sample size is small, homogeneity within *C. hominis* at the GP60 locus is unusual, as common subtype families such as Ia and Id are usually equally abundant in most developing countries (*2**,**10**,**11*). Only some industrialized nations, such as Portugal and the United Kingdom, are known to have limited heterogeneity in *C. hominis* infections in humans (*12**,**13*). The GP60 subtype IbA10G2 identified here is identical to that previously reported in the United States, the United Kingdom, Portugal, South Africa, and Peru. Another Ib subtype, IbA9G3, which was commonly reported in India, Malawi, and Australia (*4**,**8**,**10**–**12*), was not seen in the specimens from Jamaica. Likewise, most Ie infections in humans are caused by IeA11G3T3, but our study identified a less common subtype, IeA12G3T3, which was also found in specimens from New Orleans (USA) and Australia (*4*). The limited number of GP60 subtypes would suggest an epidemic mode of transmission. The extended period of specimen collections and the identification of other *Cryptosporidium* species do not indicate that a cryptosporidiosis outbreak occurred during this long study period.

All *C. hominis* specimens from Jamaica, irrespective of the GP60 subtype (IbA10G2 or IeA12G3T3), belonged mostly to 1 MLST group distinct from other subtypes from other geographic regions. These results suggest that a unique *C. hominis* parasite population is being transmitted in Jamaica. Heterogeneity was higher in the *C. parvum* population despite the smaller number of specimens that we analyzed. As with *C. hominis*, a unique population of *C. parvum* in Jamaica also seems to exist. These results further show the distinct population of *Cryptosporidium* spp. that can arise because of geographic segregation. When one considers that all *C. parvum* specimens belonged to only 1 GP60 subtype, whether the high diversity seen with the MLST is due to recent expansion of an ancestral type in the region is not clear. A study of population genetics of *Cryptosporidium* spp. from humans in Haiti showed limited multilocus subtypes in both *C. hominis* and *C. parvum*, which was interpreted as an indication for an epidemic clonal population for both species (*6*).

The occurrence of unique multilocus subtypes in both *C. hominis* and *C. parvum* populations in Jamaica that can be differentiated from other geographic regions, including the nearby United States, is notable and requires further investigations. The distinction is important in mapping the transmission of the parasite, especially where tracking infection pathways is necessary, such as in investigations of outbreaks and traveler’s diarrhea. The fact that *C. hominis* from different areas may have an identical GP60 subtype but a different MLST subtype is also noteworthy, as some *C. hominis* subtypes, such as IbA10G2, are commonly associated with water-borne outbreaks in industrialized countries. The clinical manifestations of the GP60 subtypes in relation to the distinct MLST from different geographic areas needs to be studied further. This would clarify how geographic segregation in *Cryptosporidium* MLST subtypes relates to GP60 subtypes that have various pathogenicities.
